# Exploring the Impact of Contaminants of Emerging Concern on Fish and Invertebrates Physiology in the Mediterranean Sea

**DOI:** 10.3390/biology12060767

**Published:** 2023-05-24

**Authors:** Federica Impellitteri, Cristiana Roberta Multisanti, Polina Rusanova, Giuseppe Piccione, Francesca Falco, Caterina Faggio

**Affiliations:** 1 Department of Veterinary Science, University of Messina, Viale Giovanni Palatucci snc, 98168 Messina, Italy; federica.impellitteri@studenti.unime.it (F.I.); gpiccione@unime.it (G.P.); 2 Department of Chemical, Biological, Pharmaceutical, and Environmental Sciences, University of Messina, Viale Ferdinando Stagno D’Alcontres, 31, 98166 Messina, Italy; cristiana.multisanti@studenti.unime.it; 3 Department of Biological, Geological and Environmental Sciences (BiGeA)—Marine Biology and Fisheries Laboratory of Fano (PU), University of Bologna, 61032 Bologna, Italy; polina.rusanova@irbim.cnr.it; 4 Institute for Marine Biological Resources and Biotechnology (IRBIM)—CNR, L. Vaccara, 91026 Mazara del Vallo, Italy

**Keywords:** bioindicators, emerging contaminants, environmental toxicity, Mediterranean Sea

## Abstract

**Simple Summary:**

Consumption and excessive use of substances or items that are taken for granted in our daily lives such as personal care products, plastic objects, and medicines inevitably lead to their release into marine and freshwater systems with, unfortunately, potentially devastating consequences over time. For this reason, it is of crucial importance that the scientific community, as well as society, is aware of the environmental problems linked mainly to the release of contaminants into the sea. In this context, the Mediterranean Sea is a special biodiversity hotspot of marine fauna and flora. Nevertheless, it is becoming a source of concern due to the presence of emerging pollutants. In the present paper, the specific focus on the catshark *Scyliorhinus canicula*, as a vertebrate species, and on the Mediterranean mussel *Mytilus galloprovincialis*, as an invertebrate, lies in their ability to provide appropriate information about the health conditions of their surrounding environment. The studies reported on this topic show that it is rather evident that entire aquatic ecosystems suffer from anthropogenic pollution. Therefore, this review aims to collect and demonstrate its effects on the environment and organisms’ health.

**Abstract:**

In this historical context, the Mediterranean Sea faces an increasing threat from emerging pollutants such as pharmaceuticals, personal care products, heavy metals, pesticides and microplastics, which pose a serious risk to the environment and human health. In this regard, aquatic invertebrates and fish are particularly vulnerable to the toxic effects of these pollutants, and several species have been identified as bio-indicators for their detection. Among these, bivalve molluscs and elasmobranchs are now widely used as bio-indicators to accurately assess the effects of contaminants. The study focuses on the catshark *Scyliorhinus canicular* and on the Mediterranean mussel *Mytilus galloprovincialis*. The first one is a useful indicator of localised contamination levels due to its exposure to pollutants that accumulate on the seabed. Moreover, it has a high trophic position and plays an important role in the Mediterranean Sea ecosystem. The bivalve mollusc *Mytilus galloprovincialis*, on the other hand, being a filter-feeding organism, can acquire and bioaccumulate foreign particles present in its environment. Additionally, because it is also a species of commercial interest, it has a direct impact on human health. In conclusion, the increasing presence of emerging pollutants in the Mediterranean Sea is a serious issue that requires immediate attention. Bivalve molluscs and elasmobranchs are two examples of bio-indicators that must be used to precisely determine the effects of these pollutants on the marine ecosystem and human health.

## 1. Introduction

Environmental quality standards and emerging contaminants are linked. Data on a novel drug’s environmental chemistry, ecotoxicology, human toxicity, and epidemiology grow as it begins to rise concerns. Finally, this prompts the creation of environmental standards or criteria by the governments to ensure proper protection. Directly measures on invertebrate models and fish are the best way to study the toxic effects of some emerging contaminants on marine organisms. Additionally, by utilising some models, we can better appreciate the difficult effects on the environment and conservation. 

The Mediterranean Sea is a unique and diverse marine ecosystem, home to a variety of invertebrates, fish, and other aquatic species. Recently, however, the Mediterranean Sea has become a source of concern due to the presence of emerging pollutants, including pharmaceuticals and personal care products, heavy metals, pesticides, and microplastics [1,2,3,4,5,6,7].

Many of these substances reach coastal and marine waters through direct discharge or river transport due to human activities, including the use of pharmaceuticals on farms, the discharge of untreated wastewater, and the release of heavy metals and pesticides by industry. All these voluntary or involuntary patterns of release of toxic substances into the environment can threaten aquatic ecosystems [8]. 

Generally, all these pollutants are highly toxic to marine life, through studies showing that they are able to lead to physiological changes, reproductive issues, and even mortality. In the Mediterranean, numerous species are indicated as bio-indicators for the detection of toxic substances in the environment [9,10]. Most of them belong to the invertebrates, such as the mollusc *Mytilus galloprovincialis*, which, as a benthic filter-feeding organism, is reported to be one of the most appropriate bio-indicators. Indeed, they have been widely used to learn about the effects related to pharmaceuticals and personal care products. The effects of two toxicants, i.e., Acetylsalicylic acid (a drug commonly used as an analgesic) and Quaternium-15 (a surfactant easily found in soaps) were evaluated by considering the alteration on the digestive gland cells functionality of *Mytilus galloprovincialis* from the Ionian coast of the Strait of Messina [11]. Particularly, these studies showed a decrease in the normal cellular capacity on the Regulatory Volume Decrease (RVD) test. Cell volume regulation is essential for maintaining the physiology of cell metabolism steady and is involved in specific vital functions such as maintaining the correct cellular pH and ensuring the correct conditions for membrane transport [12]. Particularly, cell volume regulation involves the process of regulatory volume decrease (RVD) response, which entails the efflux of ions and osmolytes from the cell to reduce its volume. However, exposure to pollutants and other environmental stressors can result in damage to the cell membrane and its associated proteins, leading to a loss of the RVD response. When this occurs, cells are unable to return to their original volume, which can disrupt normal cellular processes and contribute to physiological alteration progression. This mechanism, via the analysis of this capacity, reflects the cellular physiological state of the animal and is particularly valued as a method for testing model organisms such as *Mytilus galloprovincialis* for the toxicity of numerous contaminants [13].

Mediterranean Sea invertebrates and fish are particularly vulnerable to the effects of pesticides, as demonstrated through a study on the haematological parameters of the freshwater catfish *Mystus keletius* [14].

In order to measure the impact of these emerging pollutants on invertebrates and fish in the Mediterranean Sea, researchers used a range of methods to assess the levels of pollutants in the water and the body of studied species in the Mediterranean region. These include chemical water analysis and monitoring the presence of pollutants in the tissues of invertebrates and fish. In addition, researchers also examined the reproductive success of Mediterranean Sea invertebrates and fish, and their ability to survive in polluted water. The results of these studies have been mixed, with some species showing signs of physiological stress, while others appear to be relatively unaffected by the presence of emerging pollutants [15]. 

However, it is clear that the presence of these pollutants is having a significant impact on the health of the fish and invertebrates in the Mediterranean Sea, and further research is required to understand the full extent of the damage caused. It is important to understand the long-term effects of the pollutants on species’ health, as well as the potential bioaccumulation of the pollutants in the tissue of fish. For instance, two commercial fish from the Mediterranean Sea were used in a comparison of the bioaccumulation of total aliphatic hydrocarbons (TAH), and it was discovered that each fish had a unique capacity for bioaccumulation that was correlated with the number of fatty acids in the liver [16]. In conclusion, researchers should prioritize studying the effects of emerging pollutants on Mediterranean Sea invertebrates and fish due to their reported association with a variety of adverse health effects in these organisms.

## 2. Materials and Methods

The data presented in this review were obtained through a careful search of several search engines commonly used for scientific research, including Google Scholar https://scholar.google.it (accessed on 10 March 2023); Pubmed, https://pubmed.ncbi.nlm.Nih.gov (accessed on 11 March 2023); Web of Science, https://clarivate.com/webofsciencegroup/solutions/web-of-science/ (accessed on 13 March 2023); and Scopus, https://www.scopus.com/home.uri (accessed on 13 March 2023). 

The search criteria included Boolean structure using “and” or “+” to obtain better results. During the search, the following keywords were used: “pollutant”, “emerging pollutants”, “impact”, “*Scyliorhinus canicula*”, “biomarker”, “daily care”, “shark”, “fish”, “plastic”, “aquatic invertebrates”, “*Mytilus galloprovincialis*”, and the articles were selected based on the presence of the keywords in the title or abstract. The search also followed a temporal order in which the most recent papers were favoured.

The information has been classified into two tables. The first concerns the direct influence on organisms. The second concerns the different emerging contaminants and their presence in different species, with emphasis on the species *Mytilus galloprovincialis* as an invertebrate model, and *Scyliorhinus canicula*, as a vertebrate species. 

## 3. Bivalve Molluscs and Elasmobranchs as Suitable Indicators of Emerging Pollutants

### 3.1. Bivalve Molluscs

Among model organisms commonly used in experimental research, invertebrates play a crucial role, due to their intermediate position in food chains [17]. Among bivalve mollusc species, *M. galloprovincialis* is highly valued for its crucial role in environmental monitoring programmes as an early indicator of pollution [18,19]. These mussels have become a cornerstone of such programmes [20,21] due to their unique ability to accumulate and reflect the presence of contaminants in their tissues. As filter feeders, they actively extract water from their surroundings, inadvertently ingesting various substances, including pollutants. Consequently, contaminant levels detected in mussel tissues provide valuable information on the quality of the surrounding aquatic environment [22]. Furthermore, the use of mussels as bio-indicators offers several advantages. Their stationary nature allows for convenient and economical sampling, making them suitable for long-term monitoring. In addition, the ability of mussels to integrate the effects of multiple contaminants over time provides a comprehensive perspective on the overall state of environmental pollution.

Due to their capillary distribution, high filtration rate and long-life span, mussels serve as effective sentinels to assess the health of marine and freshwater ecosystems [23]. Their regular monitoring allows researchers and environmental agencies to detect and assess the presence of emerging pollutants, such as heavy metals, pesticides, and pharmaceutical residues, which may pose a risk to human and ecosystem health [24].

### 3.2. Elasmobranchs 

In recent years, researchers have become increasingly interested in studying the impact of various types of environmental pollutants such as heavy metals, Dichlorodiphenyltrichloroethane (DDT), polychlorinated biphenyls (PCBs), microplastics, and personal care products on marine organisms, especially Elasmobranchs. In addition, one of the interesting aspects, because more attention has been focused on other vertebrate groups than the Elasmobranchs group, is that many Elasmobranch species already have strong anthropogenic influence, such as unrestricted illegal fishing activity [25]. As a result of this non-ecological-friendly way of using biological recourse, one in four of these Elasmobranch species (with special attention on the largest sharks and rays) are threatened with extinction [26]. 

Indeed, to confirm this, Falco et al. [27] in their studies conducted on the blood biomarkers of the catshark *Scylyorinus canicular* and suggested that over-exploitation can be detrimental to the physiological system of the aforementioned species. Moreover, Elasmobranchs have a unique physiology that can make them particularly vulnerable to environmental pollutants due to their slow growth, low reproductive rate, long lifespan, and occupancy of top trophic positions. These factors make them more susceptible to the accumulation of pollutants over time, which can have negative impacts on their health, overall survival and in general the population. For our research, we chose one of the most common species of subclass Elasmobranchii, which includes sharks, rays, and skates [28]. 

Our focus is on the shark species *Scyliorhinus canicula*. Our choice is explained by a few reasons. Firstly, *S. canicula* is a relatively small, non-migratory species that spends much of its life on the seafloor. This means that it is exposed to pollutants that accumulate on the seafloor, making it a useful indicator of localized pollution levels [29,30]. Secondly, the small-spotted catshark—*S. canicula* has a high trophic position and an important role within marine ecosystems and in the Mediterranean Sea. *S. canicula* is a mesopredator, which impacts the dynamics and stability of marine systems, by connecting different food webs and trophic levels in aquatic ecosystems. The diet of *S. canicula* consists of several other species, including crustaceans, molluscs, and other fish. The latter could account for the bioaccumulation of pollutants in its tissues over time, making it a useful bio-monitor for longer-term exposure to sea pollutants. One of the important parameters for choosing *S. canicula* as an indicator of pollutants [31,32,33] is that it is a hardy and resilient species that can tolerate a range of different environmental conditions. This means that it can be found in a range of different habitats and is less likely to be affected by natural fluctuations in the environment, making it easier to identify changes in pollutant levels [34]. 

## 4. An Overview of Emerging Contaminants Toxicity 

The topic of emerging contaminants and their toxicity is of great significance and continues to attract the attention of the scientific community. In recent years, a significant number of articles have been published on this subject in different scientific journals. According to our search on Google Scholar, it has been found that approximately 20,900 research papers have been published on emerging contaminants in water over the last ten years, indicating the significant attention and research dedicated to this topic over the past decade.

As shown in Table 1, different emerging contaminants may have different effects depending on their type and chemical composition, although the results of numerous studies conducted on their toxicity represent them as harmful substances to both ecosystems and organisms. 

In this section, the effects of the relevant contaminants on the model organisms described will be addressed, in order to understand their mode of action within organisms, as well as their consequences at population and ecosystem levels. Moreover, Table 2 contains many of the emerging contaminants reported in this review also highlighting the specific part of the Mediterranean Sea to which each study refers.

### 4.1. Microplastics

The production, use, and mass consumption and disposal of plastic objects are causes of environmental pollution, especially in aquatic habitats. Once plastic wastes are released into the environment, they are subjected to mechanical forces and sunny radiations resulting in the formation of microplastics [62,63,64,65]. Indeed, microplastics are small plastic particles less than 5 mm in diameter [66,67,68] that enter the ecosystem as a result of the breakdown of large plastic particles or the direct release of small plastic particles by climate and human activities [69]. Due to size, shape, and composition, microplastics can be confused with food from aquatic animals actively feeding, whereas they are naturally internalized by filtering-feeding organisms [70]. For example, nanoplastics are even smaller plastic particles than microplastic (which measure less than 100 nanometers in size) and because of their tiny size, have the potential to enter living organisms at the cellular level. Nanoparticles possess distinctive physicochemical properties that can lead to unpredictable interactions with cells and tissues [71]. Due to their comparable size, fabricated nanoparticles may directly interact with cellular molecular organelles and macromolecules, revealing potential nano-bio interface effects [22]. For these reasons, microplastics and nanoplastic can easily reach the top of trophic chains through bioaccumulation and biomagnification processes [62,72].

The Mediterranean Sea accumulates an estimated annual amount of 150 to 610 thousand tonnes of plastics (with an average of 229 thousand tonnes), of which 94% consists of microplastic debris and 6% are microplastics, as stated by IUCN [73]. Plastic particles with a density lower than seawater (which includes most synthetic polymers) typically float on the water’s surface, whereas those with higher density sink and accumulate on the seafloor. However, buoyant particles may also sink due to biofouling and particle adherence, and this can significantly impact the distribution of plastic pollution in the ocean. The impacts of microplastics by ingestion, smothering, or entanglement have been well documented for a variety of marine species, which has led to the consideration of plastics as hazardous materials [33,48].

For example, in a study conducted by Álvarez-Ruiz et al. [24]., the bioaccumulation of 20 emerging contaminants, including pharmaceuticals, pesticides, and perfluoroalkyl substances (PFAS), in the mussel *Mytilus galloprovincialis* with or without the presence of microplastics was assessed. The results revealed that some contaminants accumulated in the visceral mass and haemolymph of the mussels and that the presence of microplastics facilitated higher bioconcentration factors and slower elimination rates. Another study conducted by the research group of Trestrail et al. [74] examined the impact of spherical microplastics ingested by the mussel *M. galloprovincialis* on digestive enzyme activities. This resulted in alterations that may affect the mussels’ acquisition of energy from food and deplete their energy reserves.

In addition to the mentioned studies, another research conducted by Mancia et al. [47] assessed plastic ingestion by the catshark *Scyliorhinus canicula* in the Mediterranean Sea and analyzed the expression levels of immune-related genes. The findings revealed that microplastics were widely ingested, and macroplastics were present in approximately 18% of the specimens. Moreover, specimens with macroplastic ingestion exhibited a significant increase in the expression of immune genes, indicating that plastic pollution represents an emerging threat to catsharks and the Mediterranean food web.

### 4.2. Personal Care Products 

The current society has become accustomed to using a wide range of personal care products (PCPs) [75,76,77,78,79]. The class of PCPs includes a variety of products such as body creams and soaps, sunscreens, exfoliants, shampoos, detergents, perfumes, cosmetics, and toothpaste [80,81], according to the US Environmental Protection Agency (US EPA). Nevertheless, the removal of PCPs from wastewater is not always successful, and their concentrations, in both marine and freshwater environments, have been found to be in the range of ng/L–μg/L, which may not seem that high, but still biologically relevant [81]. For this reason, PCP-related substances can lead to potential risk as a source of stress for marine species and ecosystems, affecting different biological levels from cellular interactions to the whole ecosystem, as shown in Table 1. An investigation of the toxicity of sodium lauryl sulphate (SLS), an anionic surfactant used as an emulsifying detergent, conducted on the Mediterranean mussel, *M. galloprovincialis*, and reported by Freitas et al. [82] revealed that the substance had a wide range of effects on the organism. There was a noticeable reduction in respiration rate, which led to a loss in filtration capacity and had negative consequences on the physiological development of organisms. Furthermore, SLS bioaccumulation in mussels affected their metabolic performance and a reduction in the efficiency of natural antioxidant mechanisms. SLS toxicity was also evaluated on the antioxidant system in primary hepatocyte cultures of Van fish (from Lake Van, Turchia). Again, the results showed alterations in the enzymatic activity of the antioxidant defense system [83]. Briefly, changes in the antioxidant defense system, lead to an imbalance between the free radicals produced by cellular metabolic reactions (known as cellular respiration) and the substances used by the body to counteract their effects. This imbalance in turn leads to the expression of oxidative stress. Lastly, it has been observed that modifications brought about by PCPs at the cellular and sub-cellular levels can have far-reaching consequences, which may culminate in effects on populations, including entire communities and thus marine ecosystems [84]. Little is currently known about how personal hygiene items affect elasmobranchs, in particular, *S. canicula*. 

A study on the Brazilian guitarfish, *Pseudobatos horkelii* [85] showed how PCPs, particularly legacy pollutants, can influence maternal load and transmit it to the offspring.

Indeed, they hypothesized that the highest transfer rates of methylparaben to the offspring were 6%, due to the presence of this pollutant in the maternal uterus samples. Additionally, a prior study by Martins et al. [86] on the subject of the aforementioned species, as well as other guitarfishes revealed that it is possible for PCPs contaminants to be transferred from mothers to offspring during vitellogenesis when yolk precursors are transferred to ovarian follicles. As a result, the contaminated yolk may be a significant source of contamination for these organisms. 

A recent study [87] conducted by the same group investigated the effects of prenatal exposure to contaminants including PPCs in embryos of Brazilian guitarfish. The results suggest that prenatal exposure to contaminants may impact redox status and lead to oxidative damage in embryos.

### 4.3. Pharmaceuticals 

Nowadays, drug consumption is also increasing due to the growing demand for various applications related to chronic diseases, but also, for example, to ageing or always much-increasing demand for farmed food without neglecting or forgetting all agricultural activities [88,89]. The consumption of pharmaceuticals by humans leads to the excretion of their metabolites, which are subsequently released into the environment [42]. Pharmaceuticals, as well as some personal care products, can act as endocrine disruptors. In other words, they can activate or inhibit specific signalling pathways that are downstream of hormonal activity, acting as “hormone mimics” [90]. This kind of interference could lead to damage to the immune system, reducing its effectiveness, hormonal imbalances, and other adverse effects in organisms exposed to these compounds. In this context, according to Pagano et al. [11], the toxicity of anti-inflammatory drugs, i.e., acetylsalicylic acid (ASA) on *M. galloprovincialis* caused alterations in hepatocytes volume regulation, which also resulted in inflammation at the histological level. The gills were also damaged by exposure to ASA and showed numerous alterations such as infiltration of haemocytes, which are responsible for the first defense of the organism against pathogens and xenobiotics. The toxicity due to waterborne antidepressant presence in aquatic environments on non-target organisms living in surface waters was also assessed. Indeed, as stated by Sehonova et al. [42], the investigation conducted into the effects of selective serotonin and selective serotonin-noradrenalin reuptake inhibitors on invertebrates, amphibians, and fish showed that the first tested drug negatively interfered with the behaviour, reproduction, and development of both invertebrates and fish. However, concerning the second antidepressant tested, it was shown not only to affect fish behaviour but also lead to an increase in fish mortality associated with developmental delay, and morpho-pathological alterations in the brain, heart, and cephalic and caudal kidney. Finally, alterations in the natural antioxidant system and an increase in lipid peroxidation were also detected, indicating the high toxicity of the drugs even at the lowest concentrations.

Sharks, compared to bony fish, have received little attention with regard to drug exposure. Information on the effect of drugs on elasmobranch species is very scarce, and we would like to note the gap on this point. For this reason, we chose to consider reporting in this review the literature studies on elasmobranchs conducted in recent years. The main works on the effect of drugs have been conducted on river elasmobranchs, such as those on the absorption of any active pharmaceutical ingredient of human drugs (e.g., citalopram, fluoxetine, fluvoxamine, paroxetine, sertraline, venlafaxine) that were examined and compared in the plasma of neonatal bull sharks (*Carcharhinus leucas*) residing in pristine tributaries (Myakka River) and in tributaries with sewage (Calohosaatchee River) of the Charlotte Harbor estuary in Florida. Gelsleichter and Szabo [91] showed that in the latter case, the drugs can accumulate and put the *C. laucas* population at risk, although the effect remained undiscovered. Instead, Martins et al. [85] demonstrated that the transfer of diclofenac from a maternal uterus to the offspring can occur at a rate of 27%.

### 4.4. Pesticides 

The growing society’s demand for plant products has also led to an increase in the use of useful products to safeguard crops from pathogens and predators, i.e., pesticides [92,93,94]. Within the class of pesticides, insecticides, fungicides, and herbicides, which may have a specific or broad-spectrum mode of action, occupy a key position. These compounds are easily able to reach aquatic ecosystems through several pathways, including wastewater and the atmosphere, interacting with the environment and the organisms that inhabit it [95]. 

In this context, the question that naturally arises is: why are aquatic invertebrates so sensitive, for example, to insecticides? The answer lies in their proximity to insects, as they share not only the same neurological and respiratory mechanisms but also the same detoxification system, which in both cases is not enough efficient in degrading pesticides [96]. 

Particularly relevant is a study conducted by Bado-Nilles et al. [97], in which the effects of a mix of 14 pesticides on the oyster *Crassostrea gigas* haemocytes were investigated. In addition, Matozzo et al. [98] also observed that *Ruditapes philippinarum* exposure to different concentrations of glyphosate (a non-selective herbicide) had a significant impact on its haemocytes. Indeed, the results showed that after 7 days of exposure to the pollutant, the total number of haemocytes decreased, and their volume increased significantly in the specimens exposed to 100 and 1000 µg/L of glyphosate. Belonging to the class of neonicotinoids is thiacloprid, a neuroactive insecticide whose chemical structure resembles that of nicotine. As neonicotinoids are competitors of acetylcholine, their action mechanism involves disrupting signal transmission. An investigation of thiacloprid toxicity on haemocytes was conducted by long-term exposure of *M. galloprovincialis* to different concentrations of the compound. The results showed that both concentrations of 1.5 and 10 µg L^−1^ caused a significant decrease in Cl^−^ and K^+^ and a significant increase in glucose content [42].

Some pollutants belonging to the class of pesticides are also known as endocrine disruptors and have been shown to alter the expression of hormones such as 17β oestradiol, progesterone, testosterone, and vitellogenin. The effects of nonylphenol (NP), an environmental pollutant similar to oestrogen, have been evaluated on the synthesis of vitellogenin of adult male cartilaginous fish “*Torpedo marmorata*” [99]; the latter studies showed that injecting *T. marmolata* males with nonylphenol in the liver and kidney resulted in the presence of VTG; this is an extraordinary factor given that this lipophosphoglycoprotein is physiologically induced by oestrogens only in females of oviparous and ovoviviparous vertebrates.

Marsili et al. [100] proposed skin biopsies to assess the toxicological effects of this organochlorine (OC) and polycyclic aromatic (IPA) pollutants in *Carcharodon carcharias* from the South African coast. These studies showed that high concentrations of OC and IPA caused significant loading responses of cytochrome P4501A (CYP1A) in the animal; in addition, vitellogenin and radiated zone proteins were also affected by these contaminants. These latter protein biomarkers were found in the gonads of immature males and females.

### 4.5. Heavy Metals 

Heavy metal pollution by anthropogenic activities is a major concern due to its impact on the environment and aquatic organisms [101,102]. Anthropogenic sources of metals include wastewater, traffic emissions, coal and oil combustion, industrial production, and many others [103]. Aquatic organisms counteract continuously with heavy metal ions also ingesting them from their diet, and their removal from tissues is not always constant as several factors come into play, such as time of exposure to the metal, temperature, metabolic activity, and metal chemistry. At low concentrations, most of these metals are essential for life, as they contribute to bone and shell or muscle development, as well as being cofactors in many biochemical reactions within the body. In the case of invertebrate organisms, the accumulation of heavy metals can directly impact the central nervous system and disrupt homeostasis. Mussels, being filter feeders, are particularly prone to accumulating heavy metals in their tissues due to their feeding habits and prolonged exposure to contaminated water. These effects can manifest in malformations, impaired growth, and reproduction, weakened immune responses, and even mortality [104,105,106].

In addition, sharks showed to be particularly sensitive to heavy metals, such as mercury, lead, and cadmium. Typically, heavy metals tend to accumulate in the liver, kidneys, muscle tissue, and blood of fish [107] as well as in sharks [108]. These metals can also accumulate in the shark’s skin and cartilage, depending on the species and the type of metal [108]. The liver is often the primary site of heavy metal accumulation in sharks because it plays a critical role in the detoxification and processing of these metals. The kidneys also play a similar role in removing toxic metals from the shark’s body. However, in some cases, the concentration of heavy metals in the muscle tissue can also be high enough to make the consumption of shark meat potentially hazardous to humans. According to Wosnick et al. [32], *S. canicula* exposure to heavy metals could lead to alterations in hepatic markers with the bioaccumulation of Co, Fe, and Hg in the liver of sharks, in urea and lactate with the bioaccumulation of Fe and Hg in the gills. Moreover, in sharks, the rectal gland plays an important role in shark osmoregulation and associated homeostatic balance. In this context, alteration in phosphorus content with the bioaccumulation of Co, Mn, and Hg in the rectal gland of sharks was also observed. 

## 5. Research Gaps and Opportunities

Scientific progress in the field of environmental pollution studies is of paramount importance as, unfortunately, recent years are witnessing an increase in environmental pollution with certain future consequences on human health as well. Precisely for this reason, we believe that disseminating information and making the community increasingly aware of the risks linked to pollution is of fundamental importance. Indeed, toxicological studies are part of the priorities of those who wish to protect the environment, organisms, and also human health, in accordance with the “One Health” approach. 

One point that must be borne in mind is that different contaminants will be present in aquatic ecosystems at the same time and as mixtures, so they may have synergistic, antagonistic, or neutral effects on each other toxicity. Briefly, a synergistic effect entails an increase in the effect of the toxicant on the established endpoints, whereas an antagonistic effect leads to a reduction in the contaminant toxicity with direct results on the biomarkers analyzed. Therefore, toxicological studies of aquatic environment contaminants require knowledge of the impact of individual contaminants, as well as the effects resulting from their interactions.

Moreover, although there is a growing general interest in the scientific community and more data are available in the literature for some of the emerging contaminants reported in this review, according to our research, effects related to personal care products and pharmaceuticals are still under-investigated, and fairly recent data of heavy metals detection on model organisms such as *Mytilus galloprovincialis*, in the Mediterranean Sea, are not still available. 

Of critical importance also turns out to be recognizing these effects on a large ecological scale, from the individual organism to the biodiversity-level interaction. Indeed, the health of an ecosystem depends on the interaction of multiple physical, chemical, and biological factors. Particularly, ecosystem processes, such as productivity and nutrient recycling, are directly related to the functional diversity of biotic communities, which in turn are determined by species biodiversity. Nevertheless, species biodiversity can be altered in response to pressures by environmental changes, and this is directly reflected in ecosystem processes’ functionality [109]. 

For these reasons, in accordance with the needs arising from the adverse effects of emerging contaminants, it is of paramount importance for the scientific community to continue in this direction carrying out investigations related especially to aquatic environments, which play the role of one of the greatest pollutant sinks, with a special focus on the effects at different ecological levels, from the individual organism to the influence on biodiversity.

## 6. Conclusions

The research conducted highlighted how starting from our daily habits, as well as societal and industrial development, it is made possible to negatively interfere at different biological and ecological levels. A crucial point is to make today’s society aware of the impact it has on its surroundings, which is reflected both in animal organisms and their biodiversity, a very important index of health in any environment, as well as indirectly on our own health. Therefore, the purpose of this review is to provide information highlighting the toxic effects on suitable model organisms in order to raise awareness of the responsible and conscious use of the major part of the products we mentioned whose use is now extremely common. From the analysis carried out in this review, it is crucial to monitor contamination levels and implement effective management strategies to prevent further damage to the environment and vulnerable species. It is imperative that action is taken to mitigate the impact of emerging pollutants on the Mediterranean Sea and its inhabitants. Our objective was to describe the type of difference between two different organisms used as models, albeit belonging to different levels of the food web. In this review, it was highlighted how all effects are refined at the level of the physiological system; if we do not pay sufficient attention to how to defend the environment from contaminants, we risk incurring serious diseases and subsequently human extinction.

## Figures and Tables

**Table 1:** Effects of emerging contaminants on several species identified as model organisms.

Type of Pollutant	Impact on Aquatic Organisms	References
**DDTs and toxic evaluation of polychlorinated biphenyls (PCBs)**	Accumulation profile analysis of individual PCB congeners and the levels of DDT and its metabolites in the liver of *S. canicula*, from the Mediterranean Sea.	Storelli et al. [35]
**Microplastic particles and textile microfibers**	Occurrence of MPs in Adriatic food webs in different species (several fish and invertebrates including *M. galloprovincialis*)	Avio et al. [36]
**MPs**	MPs influence on marine organisms	Baldwin et al. [37]
**MPs**	Plastic impact on sharks and rays.	Lipej et al. [38]
**MPs**	Analysis of the different influence of water depth, feeding habits and diets on microplastics accumulation.	Aiguo et al. [39]
**MPs and NPs**	Toxic effects and bioaccumulation on several aquatic species identified as suitable bioindicators of microplastic pollution.	Multisanti et al. [22]
**MPs in cosmetics**	Statistical data on MPs in cosmetics.	Guerranti et al. [40]
**Pharmaceuticals and Personal care products**	Deep explanation about different chemical compositions: pharmaceuticals and personal care products.	Salimi et al. [41]
**Pharmaceuticals**	Analysis on the impact of tricyclic antidepressants on non-target organisms showed hearth, brain, and cranial and caudal kidney damage; oxidative damage of lipids and also a significant increase in mortality.	Sehonova et al. [42]
**Pesticide (fungicide)**	Study of the potential risks of a fungicide in the model organism *M. galloprovincialis*, mainly through its bioaccumulation, and the alteration of fundamental physiological processes.	Tresnakova et al. [13]
**Pesticide (insecticide)**	Evaluation of toxicity due to NeemAzal T/S exposure on early-life stages of common carp (*Cyprinus carpio* L.) showed gills histophatological changes linked to a significant increase in glutathione oxidase, glutathione S-transferase activity and also an increase in oxidised lipids. Moreover, the exposure also induced slow hatching and an increase in mortality.	Chromcova et al. [43]

**Table 2:** Occurrence and effects of emerging contaminants in relation to samples of species from different geographical areas of the Mediterranean Sea.

Geographical Location	Kind of Pollutant	Species	Influence	References
**Strait of Messina, central Mediterranean Sea**	Polystyrene microplastics	*Mytilus galloprovincialis*	Insights into early mechanisms of toxicity of polystyrene MPs in mussels. Disorders in osmoregulation, energy and protein metabolism, and oxidative stress were detected.	Cappello et al. [44]
**North Adriatic Sea**	3 μm polystyrene microplastics	*Mytilus galloprovincialis* (larvae)	Gene expression of genes involved in growth and adaptation mechanisms, due to exposure to polystyrene MPs, was altered.	Capolupo et al. [45]
**Black Sea, Aegean, and the Marmara Sea**	Microplastic particles	*Mytilus galloprovincialis*	Occurrence of MPs in all the samples from each geographic area analysed.	Gedik and Eryaşar [46]
**Southern region of the central Mediterranean Sea.**	Plastics injection	*Scyliorhinus canicula*	The presence of plastics, especially macroplastics in the gastrointestinal (GI) tract, was correlated with an increase in the hepatosomatic index and an increased expression of 3 essential immune system genes. It is hypothesized that these effects are induced by additives that are leaching from the ingested plastics. Moreover, plastic particles also act as endocrine disruptors.	Mancia et al. [47]
**Portuguese coast**	Plastic injection/ particles and fibers	*Scyliorhinus canicula*	Depending on the size of the animal, ingested plastic fragments can be small enough to be expelled from the organism through faeces, but larger fragments may be retained in GI tract, causing a false sense of satiety. This paper showed that pelagic species ingest more particles, whereas benthic species ingest more fibres (in relationship to the presence of high quantities of fibres on the seabed).	Neves et al. [48]
**Tyrrhenian Sea**	Plastic injection	*Scyliorhinus canicula*	MPs occurrence in sharks’ GI tract. The main part of particles detected were dark-colored fibers (blue or black) in the size range of 100 μm–330 μm and in high percentages recorded in all three examined species, in high percentages.	Valente et al. [49]
**Bay Biskay**	Plastic injection	*Scyliorhinus canicula*	High percentage of MPs occurrence.	López-López et al. [50]
**Spanish Atlantic and Mediterranean coasts**	Plastic injection	*Scyliorhinus canicula*	17% of sharks analysed were found to contain MPs in their stomach.	Bellas et al. [51]
**The west coast of Mallorca and in the Mallorca Channel**	Plastic injection	*Scyliorhinus canicula*	This paper demonstrated that increasing plastic pollution is related to the increase in water depth, which possibly indicates that plastic pollution is more dependent on depth than spatial coverage.	Alomar et al. [52]
**Scotland**	Concentration and biomagnification of PCBs and PBDEs	*Scyliorhinus canicula*	All marine mammals, demersal, and pelagic fish had detectable PCBs in their tissues.	Madgett et al. [53]
**Tipaza, Algeria**	Mixture of Cd, Zn, Cu	*Mytilus galloprovincialis*	Short-term sublethal effects of cadmium (Cd), zinc (Zn), and copper (Cu), carried out via the bioaccumulation. The results revealed a high mortality in mussels exposed to the lowest concentrations of Cu. However, Cd and Zn exposure did not induce a high mortality.	Boudjema et al. [54]
**NW Mediterranean Sea & N.E Atlantic Ocean**	Metals	*Scyliorhinus canicula*	The shark *S. canicula* had the highest Zn concentrations, and this aspect has a connection because bioaccumulation capacity of Zn from seawater was particularly pronounced in this species. Thus, the peculiar metabolism of *S. canicula* regarding Zn may explain the high values measured excluding local contamination.	Mille et al. [55]
**NW Mediterranean Sea&NE Atlantic Ocean**	Metals	*Scyliorhinus canicula*	Analysis of Hg concentrations in sharks. Particularly, *S. canicula* presents the highest Hg concentrations	Chouvelon et al. [56]
**Portugal**	Metals	*Scyliorhinus canicula*	Atlantic lesser-spotted dogfish accumulate high levels of As, Zn, Fe, and Al, which were found to accumulate more in the skin than in sharks’ muscles. As levels in muscle reveal this fish unfit for feed production in the EU. Indeed, guideline limits for human consumption were overcome for Hg and As. Risk assessment of meHg and iAs levels indicate a potential risk for human health.	Marques et al. [57]
**Great Sole Bank and the Atlantic coast of the Iberian Peninsula**	Metals	*Scyliorhinus canicula*	Analysis of pollutant levels in discarded fish species by coast of the Iberian Peninsula trawlers showed Hg, Cd, and Pb concentrations in different sampled tissues.	Antelo et al. [58]
**NW French Mediterranean**	Trace elements	*Scyliorhinus canicula*	*S. canicula* presents the highest Al, As, Cd, Co, and Hg concentrations.	Bouchoucha et al. [59]
**Mediterranean**	Pollutant Pb burden	*Centroscymnus coelolepis*	Pb content of Mediterranean deep-sea *C. coelolepis* is among the lowest encountered in sharks from various habitats. Pb isotope imprints reveal that Pb is mainly from anthropogenic origin in *C. coelolepis* tissue	Veron et al. [60]
**Nigeria**	Pollutant from a solid waste dumpsite	*Clarias gariepinus*	These pollutants were found to, possibly, induce endocrine disruption.	Ibor et al. [61]

## Data Availability

No new data were created or analyzed in this study.

## References

[1] Zicarelli G., Multisanti C.R., Falco F., Faggio C. (2022). Evaluation of Toxicity of Personal Care Products (PCPs) in Freshwaters: Zebrafish as a Model. Environ. Toxicol. Pharmacol..

[2] Monique M., Giuseppe P., Francesca F., Davide D.P., Savoca S., Gioele C., Teresa R., Giovanni P., Eleonora G., Nunziacarla S. (2022). Investigating the Effects of Microplastic Ingestion in *Scyliorhinus canicula* from the South of Sicily. Sci. Total Environ..

[3] Spada L., Annicchiarico C., Cardellicchio N., Giandomenico S., DI Leo A. (2013). Heavy Metals Monitoring in Mussels *Mytilus galloprovincialis* from the Apulian Coasts (Southern Italy). Mediterr. Mar. Sci..

[4] Boldrocchi G., Spanu D., Polesello S., Valsecchi S., Garibaldi F., Lanteri L., Ferrario C., Monticelli D., Bettinetti R. (2022). Legacy and Emerging Contaminants in the Endangered Filter Feeder Basking Shark *Cetorhinus maximus*. Mar. Pollut. Bull..

[5] Brumovský M., Bečanová J., Kohoutek J., Borghini M., Nizzetto L. (2017). Contaminants of Emerging Concern in the Open Sea Waters of the Western Mediterranean. Environ. Pollut..

[6] López-Serna R., Postigo C., Blanco J., Pérez S., Ginebreda A., de Alda M.L., Petrović M., Munné A., Barceló D. (2012). Assessing the Effects of Tertiary Treated Wastewater Reuse on the Presence Emerging Contaminants in a Mediterranean River (Llobregat, NE Spain). Environ. Sci. Pollut. Res..

[7] Munschy C., Marchand P., Venisseau A., Veyrand B., Zendong Z. (2013). Levels and Trends of the Emerging Contaminants HBCDs (Hexabromocyclododecanes) and PFCs (Perfluorinated Compounds) in Marine Shellfish along French Coasts. Chemosphere.

[8] Dachs J., Méjanelle L. (2010). Organic Pollutants in Coastal Waters, Sediments, and Biota: A Relevant Driver for Ecosystems during the Anthropocene?. Estuaries Coasts.

[9] Impellitteri F., Curpăn A.-S., Plăvan G., Ciobica A., Faggio C. (2022). Hemocytes: A Useful Tool for Assessing the Toxicity of Microplastics, Heavy Metals, and Pesticides on Aquatic Invertebrates. Int. J. Environ. Res. Public Health.

[10] Pagano M., Fabrello J., Multisanti C.R., Zicarelli G., Ciscato M., Boldrin F., Giacobbe S., Matozzo V., Faggio C. (2023). A First Insight into Haemocytes of *Pinctada imbricata radiata*: A Morpho-functional Characterization. Microsc. Res. Tech..

[11] Pagano M., Savoca S., Impellitteri F., Albano M., Capillo G., Faggio C. (2022). Toxicological Evaluation of Acetylsalicylic Acid in Non-Target Organisms: Chronic Exposure on *Mytilus galloprovincialis* (Lamarck, 1819). Front. Physiol..

[12] Wehner F., Olsen H., Tinel H., Kinne-Saffran E., Kinne R.K.H. (2003). Cell Volume Regulation: Osmolytes, Osmolyte Transport, and Signal Transduction. Reviews of Physiology, Biochemistry and Pharmacology.

[13] Tresnakova N., Famulari S., Zicarelli G., Impellitteri F., Pagano M., Presti G., Filice M., Caferro A., Gulotta E., Salvatore G. (2023). Multi-Characteristic Toxicity of Enantioselective Chiral Fungicide Tebuconazole to a Model Organism Mediterranean Mussel *Mytilus galloprovincialis* Lamarck, 1819 (Bivalve: Mytilidae). Sci. Total Environ..

[14] Barathinivas A., Ramya S., Neethirajan K., Jayakumararaj R., Pothiraj C., Balaji P., Faggio C. (2022). Ecotoxicological Effects of Pesticides on Hematological Parameters and Oxidative Enzymes in Freshwater Catfish, *Mystus keletius*. Sustainability.

[15] Hodkovicova N., Hollerova A., Svobodova Z., Faldyna M., Faggio C. (2022). Effects of Plastic Particles on Aquatic Invertebrates and Fish—A Review. Environ. Toxicol. Pharmacol..

[16] Piazzese D., Bonanno A., Bongiorno D., Falco F., Indelicato S., Milisenda G., Vazzana I., Cammarata M. (2019). Co-Inertia Multivariate Approach for the Evaluation of Anthropogenic Impact on Two Commercial Fish along Tyrrhenian Coasts. Ecotoxicol. Environ. Saf..

[17] Ravi R., Athisuyambulingam M., Kanagaraj S., Tresnakova N., Impellitteri F., Viswambaran G., Faggio C. (2023). Impact of Chlorpyrifos on Cytopathological Indices in Mangrove Crab, *Episesarma tetragonum* (Fabricius). Vet. Sci..

[18] Moreira S.M., Guilhermino L. (2005). The Use of *Mytilus galloprovincialis* Acetylcholinesterase and Glutathione S-Transferases Activities as Biomarkers of Environmental Contamination along the Northwest Portuguese Coast. Environ. Monit. Assess..

[19] Lam P.K.S. (2009). Use of Biomarkers in Environmental Monitoring. Ocean Coast. Manag..

[20] González-Fernández C., Albentosa M., Campillo J.A., Viñas L., Romero D., Franco A., Bellas J. (2015). Effect of Nutritive Status on *Mytilus galloprovincialis* Pollution Biomarkers: Implications for Large-Scale Monitoring Programs. Aquat. Toxicol..

[21] Beyer J., Green N.W., Brooks S., Allan I.J., Ruus A., Gomes T., Bråte I.L.N., Schøyen M. (2017). Blue Mussels (*Mytilus edulis* Spp.) as Sentinel Organisms in Coastal Pollution Monitoring: A Review. Mar. Environ. Res..

[22] Multisanti C.R., Merola C., Perugini M., Aliko V., Faggio C. (2022). Sentinel Species Selection for Monitoring Microplastic Pollution: A Review on One Health Approach. Ecol. Indic..

[23] Curpan A.-S., Impellitteri F., Plavan G., Ciobica A., Faggio C. (2022). Review: *Mytilus galloprovincialis*: An Essential, Low-Cost Model Organism for the Impact of Xenobiotics on Oxidative Stress and Public Health. Comp. Biochem. Physiol. Part C Toxicol. Pharmacol..

[24] Álvarez-Ruiz R., Picó Y., Campo J. (2021). Bioaccumulation of Emerging Contaminants in Mussel (*Mytilus galloprovincialis*): Influence of Microplastics. Sci. Total Environ..

[25] Tiktak G.P., Butcher D., Lawrence P.J., Norrey J., Bradley L., Shaw K., Preziosi R., Megson D. (2020). Are Concentrations of Pollutants in Sharks, Rays and Skates (Elasmobranchii) a Cause for Concern? A Systematic Review. Mar. Pollut. Bull..

[26] Dulvy N.K., Fowler S.L., Musick J.A., Cavanagh R.D., Kyne P.M., Harrison L.R., Carlson J.K., Davidson L.N., Fordham S.V., Francis M.P. (2014). Extinction Risk and Conservation of the World’s Sharks and Rays. Elife.

[27] Falco F., Bono G., Cammarata M., Cavalca J., Vazzana I., Dara M., Scannella D., Guicciardi S., Faggio C., Ragonese S. (2023). Stress Related Blood Values in *Scyliorhinus canicula* as Live-Indicators of Physiological Status after Bottom Trawling Capture Activity. Comp. Biochem. Physiol. B Biochem. Mol. Biol..

[28] Rodríguez-Cabello C., Sánchez F., Fernández A., Olaso I. (2004). Is the Lesser Spotted Dogfish (*Scyliorhinus canicula*) Population from the Cantabrian Sea a Unique Stock?. Fish. Res..

[29] Esposito G., Prearo M., Renzi M., Anselmi S., Cesarani A., Barcelò D., Dondo A., Pastorino P. (2022). Occurrence of Microplastics in the Gastrointestinal Tract of Benthic by–Catches from an Eastern Mediterranean Deep–Sea Environment. Mar. Pollut. Bull..

[30] Barbieri M., Maltagliati F., Roldán M.I., Castelli A. (2014). Molecular Contribution to Stock Identification in the Small-Spotted Catshark, *Scyliorhinus canicula* (Chondrichthyes, Scyliorhinidae). Fish. Res..

[31] Kousteni V., Karachle P.K., Megalofonou P. (2017). Diet of the Small-Spotted Catshark *Scyliorhinus canicula* in the Aegean Sea (Eastern Mediterranean). Mar. Biol. Res..

[32] Wosnick N., Niella Y., Hammerschlag N., Chaves A.P., Hauser-Davis R.A., da Rocha R.C.C., Jorge M.B., de Oliveira R.W.S., Nunes J.L.S. (2021). Negative Metal Bioaccumulation Impacts on Systemic Shark Health and Homeostatic Balance. Mar. Pollut. Bull..

[33] Smith L.E. (2018). Plastic Ingestion by *Scyliorhinus canicula* Trawl Captured in the North Sea. Mar. Pollut. Bull..

[34] Muñoz-Baquero M., Marco-Jiménez F., García-Domínguez X., Ros-Santaella J.L., Pintus E., Jiménez-Movilla M., García-Párraga D., García-Vazquez F.A. (2021). Comparative Study of Semen Parameters and Hormone Profile in Small-Spotted Catshark (*Scyliorhinus canicula*): Aquarium-Housed vs. Wild-Captured. Animals.

[35] Storelli M.M., Barone G., Santamaria N., Marcotrigiano G.O. (2006). Residue Levels of DDTs and Toxic Evaluation of Polychlorinated Biphenyls (PCBs) in *Scyliorhinus canicula* Liver from the Mediterranean Sea (Italy). Mar. Pollut. Bull..

[36] Avio C.G., Pittura L., d’Errico G., Abel S., Amorello S., Marino G., Gorbi S., Regoli F. (2020). Distribution and Characterization of Microplastic Particles and Textile Microfibers in Adriatic Food Webs: General Insights for Biomonitoring Strategies. Environ. Pollut..

[37] Baldwin W.S., Bain L.J., Di Giulio R., Kullman S., Rice C.D., Ringwood A.H., van den Hurk P. (2020). 20th Pollutant Responses in Marine Organisms (PRIMO 20): Global Issues and Fundamental Mechanisms Caused by Pollutant Stress in Marine and Freshwater Organisms. Aquat. Toxicol..

[38] Lipej L., Cumani F., Acquavita A., Bettoso N. (2022). Plastic Impact on Sharks and Rays. Plastic Pollution and Marine Conservation.

[39] Aiguo Z., Di S., Chong W., Yuliang C., Shaolin X., Peiqin L., Guohuan X., Huijuan T., Jixing Z. (2022). Characteristics and Differences of Microplastics Ingestion for Farmed Fish with Different Water Depths, Feeding Habits and Diets. J. Environ. Chem. Eng..

[40] Guerranti C., Martellini T., Perra G., Scopetani C., Cincinelli A. (2019). Microplastics in Cosmetics: Environmental Issues and Needs for Global Bans. Environ. Toxicol. Pharmacol..

[41] Salimi M., Esrafili A., Gholami M., Jonidi Jafari A., Rezaei Kalantary R., Farzadkia M., Kermani M., Sobhi H.R. (2017). Contaminants of Emerging Concern: A Review of New Approach in AOP Technologies. Environ. Monit. Assess..

[42] Sehonova P., Plhalova L., Blahova J., Doubkova V., Marsalek P., Prokes M., Tichy F., Skladana M., Fiorino E., Mikula P. (2017). Effects of Selected Tricyclic Antidepressants on Early-Life Stages of Common Carp (*Cyprinus carpio*). Chemosphere.

[43] Chromcova L., Blahova J., Zivna D., Plhalova L., Casuscelli F., Tocco D., Divisova L., Prokes M., Faggio C., Tichy F. (2015). NeemAzal T/S—Toxicity to Early-Life Stages of Common Carp (*Cyprinus carpio* L.). Vet. Med..

[44] Cappello T., De Marco G., Oliveri Conti G., Giannetto A., Ferrante M., Mauceri A., Maisano M. (2021). Time-Dependent Metabolic Disorders Induced by Short-Term Exposure to Polystyrene Microplastics in the Mediterranean Mussel *Mytilus galloprovincialis*. Ecotoxicol. Environ. Saf..

[45] Capolupo M., Franzellitti S., Valbonesi P., Lanzas C.S., Fabbri E. (2018). Uptake and Transcriptional Effects of Polystyrene Microplastics in Larval Stages of the Mediterranean Mussel *Mytilus galloprovincialis*. Environ. Pollut..

[46] Gedik K., Eryaşar A.R. (2020). Microplastic Pollution Profile of Mediterranean Mussels (*Mytilus galloprovincialis*) Collected along the Turkish Coasts. Chemosphere.

[47] Mancia A., Chenet T., Bono G., Geraci M.L., Vaccaro C., Munari C., Mistri M., Cavazzini A., Pasti L. (2020). Adverse Effects of Plastic Ingestion on the Mediterranean Small-Spotted Catshark (*Scyliorhinus canicula*). Mar. Environ. Res..

[48] Neves D., Sobral P., Ferreira J.L., Pereira T. (2015). Ingestion of Microplastics by Commercial Fish off the Portuguese Coast. Mar. Pollut. Bull..

[49] Valente T., Sbrana A., Scacco U., Jacomini C., Bianchi J., Palazzo L., de Lucia G.A., Silvestri C., Matiddi M. (2019). Exploring Microplastic Ingestion by Three Deep-Water Elasmobranch Species: A Case Study from the Tyrrhenian Sea. Environ. Pollut..

[50] López-López L., Preciado I., González-Irusta J.M., Arroyo N.L., Muñoz I., Punzón A., Serrano A. (2018). Incidental Ingestion of Meso- and Macro-Plastic Debris by Benthic and Demersal Fish. Food Webs.

[51] Bellas J., Martínez-Armental J., Martínez-Cámara A., Besada V., Martínez-Gómez C. (2016). Ingestion of Microplastics by Demersal Fish from the Spanish Atlantic and Mediterranean Coasts. Mar. Pollut. Bull..

[52] Alomar C., Deudero S., Compa M., Guijarro B. (2020). Exploring the Relation between Plastic Ingestion in Species and Its Presence in Seafloor Bottoms. Mar. Pollut. Bull..

[53] Madgett A.S., Yates K., Webster L., McKenzie C., Brownlow A., Moffat C.F. (2022). The Concentration and Biomagnification of PCBs and PBDEs across Four Trophic Levels in a Marine Food Web. Environ. Pollut..

[54] Boudjema K., Badis A., Moulai-Mostefa N. (2022). Study of Heavy Metal Bioaccumulation in *Mytilus galloprovincialis* (Lamark 1819) from Heavy Metal Mixture Using the CCF Design. Environ. Technol. Innov..

[55] Mille T., Cresson P., Chouvelon T., Bustamante P., Brach-Papa C., Bruzac S., Rozuel E., Bouchoucha M. (2018). Trace Metal Concentrations in the Muscle of Seven Marine Species: Comparison between the Gulf of Lions (North-West Mediterranean Sea) and the Bay of Biscay (North-East Atlantic Ocean). Mar. Pollut. Bull..

[56] Chouvelon T., Cresson P., Bouchoucha M., Brach-Papa C., Bustamante P., Crochet S., Marco-Miralles F., Thomas B., Knoery J. (2018). Oligotrophy as a Major Driver of Mercury Bioaccumulation in Medium-to High-Trophic Level Consumers: A Marine Ecosystem-Comparative Study. Environ. Pollut..

[57] Marques A.F.S., Alves L.M.F., Moutinho A., Lemos M.F.L., Novais S.C. (2021). *Scyliorhinus canicula* (Linnaeus, 1758) Metal Accumulation: A Public Health Concern for Atlantic Fish Consumers?. Mar. Pollut. Bull..

[58] Antelo L.T., Ordóñez-del Pazo T., Lopes C., Franco-Uría A., Pérez-Martín R.I., Alonso A.A. (2016). Pollutant Levels in Discarded Fish Species by Spanish Trawlers Operating in the Great Sole Bank and the Atlantic Coast of the Iberian Peninsula. Mar. Pollut. Bull..

[59] Bouchoucha M., Chekri R., Leufroy A., Jitaru P., Millour S., Marchond N., Chafey C., Testu C., Zinck J., Cresson P. (2019). Trace Element Contamination in Fish Impacted by Bauxite Red Mud Disposal in the Cassidaigne Canyon (NW French Mediterranean). Sci. Total Environ..

[60] Veron A., Dell’Anno A., Angelidis M.O., Aloupi M., Danovaro R., Radakovitch O., Poirier A., Heussner S. (2022). Pollutant Pb Burden in Mediterranean Centroscymnus Coelolepis Deep-Sea Sharks. Mar. Pollut. Bull..

[61] Ibor O.R., Andem A.B., Eni G., Arong G.A., Adeougn A.O., Arukwe A. (2020). Contaminant Levels and Endocrine Disruptive Effects in Clarias Gariepinus Exposed to Simulated Leachate from a Solid Waste Dumpsite in Calabar, Nigeria. Aquat. Toxicol..

[62] Aliko V., Multisanti C.R., Turani B., Faggio C. (2022). Get Rid of Marine Pollution: Bioremediation an Innovative, Attractive, and Successful Cleaning Strategy. Sustainability.

[63] Auta H.S., Emenike C.U., Fauziah S.H. (2017). Distribution and Importance of Microplastics in the Marine Environment: A Review of the Sources, Fate, Effects, and Potential Solutions. Environ. Int..

[64] Cincinelli A., Martellini T., Guerranti C., Scopetani C., Chelazzi D., Giarrizzo T. (2019). A Potpourri of Microplastics in the Sea Surface and Water Column of the Mediterranean Sea. Trends Anal. Chem..

[65] Güven O., Gökdağ K., Jovanović B., Kıdeyş A.E. (2017). Microplastic Litter Composition of the Turkish Territorial Waters of the Mediterranean Sea, and Its Occurrence in the Gastrointestinal Tract of Fish. Environ. Pollut..

[66] Conkle J.L., Báez Del Valle C.D., Turner J.W. (2018). Are We Underestimating Microplastic Contamination in Aquatic Environments?. Environ. Manag..

[67] Koelmans A.A., Redondo-Hasselerharm P.E., Nor N.H.M., de Ruijter V.N., Mintenig S.M., Kooi M. (2022). Risk Assessment of Microplastic Particles. Nat. Rev. Mater..

[68] Filella M. (2015). Questions of Size and Numbers in Environmental Research on Microplastics: Methodological and Conceptual Aspects. Environ. Chem..

[69] Llorca M., Álvarez-Muñoz D., Ábalos M., Rodríguez-Mozaz S., Santos L.H.M.L.M., León V.M., Campillo J.A., Martínez-Gómez C., Abad E., Farré M. (2020). Microplastics in Mediterranean Coastal Area: Toxicity and Impact for the Environment and Human Health. Trends Environ. Anal. Chem..

[70] Ma J., Zhao J., Zhu Z., Li L., Yu F. (2019). Effect of Microplastic Size on the Adsorption Behavior and Mechanism of Triclosan on Polyvinyl Chloride. Environ. Pollut..

[71] Kik K., Bukowska B., Sicińska P. (2020). Polystyrene Nanoparticles: Sources, Occurrence in the Environment, Distribution in Tissues, Accumulation and Toxicity to Various Organisms. Environ. Pollut..

[72] Miller M.E., Hamann M., Kroon F.J. (2020). Bioaccumulation and Biomagnification of Microplastics in Marine Organisms: A Review and Meta-Analysis of Current Data. PLoS ONE.

[73] Bråte I.L.N., Blázquez M., Brooks S.J., Thomas K.V. (2018). Weathering Impacts the Uptake of Polyethylene Microparticles from Toothpaste in Mediterranean Mussels (*M. galloprovincialis*). Sci. Total Environ..

[74] Trestrail C., Walpitagama M., Miranda A., Nugegoda D., Shimeta J. (2021). Microplastics Alter Digestive Enzyme Activities in the Marine Bivalve, *Mytilus galloprovincialis*. Sci. Total Environ..

[75] Picot-Groz M., Fenet H., Martinez Bueno M.J., Rosain D., Gomez E. (2018). Diurnal Variations in Personal Care Products in Seawater and Mussels at Three Mediterranean Coastal Sites. Environ. Sci. Pollut. Res..

[76] Montes-Grajales D., Fennix-Agudelo M., Miranda-Castro W. (2017). Occurrence of Personal Care Products as Emerging Chemicals of Concern in Water Resources: A Review. Sci. Total Environ..

[77] Sadutto D., Andreu V., Ilo T., Akkanen J., Picó Y. (2021). Pharmaceuticals and Personal Care Products in a Mediterranean Coastal Wetland: Impact of Anthropogenic and Spatial Factors and Environmental Risk Assessment. Environ. Pollut..

[78] Pinheiro M., Martins I., Raimundo J., Caetano M., Neuparth T., Santos M.M. (2023). Stressors of Emerging Concern in Deep-Sea Environments: Microplastics, Pharmaceuticals, Personal Care Products and Deep-Sea Mining. Sci. Total Environ..

[79] Hawash H.B., Moneer A.A., Galhoum A.A., Elgarahy A.M., Mohamed W.A.A., Samy M., El-Seedi H.R., Gaballah M.S., Mubarak M.F., Attia N.F. (2023). Occurrence and Spatial Distribution of Pharmaceuticals and Personal Care Products (PPCPs) in the Aquatic Environment, Their Characteristics, and Adopted Legislations. J. Water Process Eng..

[80] Brausch J.M., Rand G.M. (2011). A Review of Personal Care Products in the Aquatic Environment: Environmental Concentrations and Toxicity. Chemosphere.

[81] Fisher M., MacPherson S., Braun J.M., Hauser R., Walker M., Feeley M., Mallick R., Bérubé R., Arbuckle T.E. (2017). Paraben Concentrations in Maternal Urine and Breast Milk and Its Association with Personal Care Product Use. Environ. Sci. Technol..

[82] Freitas R., Silvestro S., Coppola F., Costa S., Meucci V., Battaglia F., Intorre L., Soares A.M.V.M., Pretti C., Faggio C. (2020). Toxic Impacts Induced by Sodium Lauryl Sulfate in *Mytilus galloprovincialis*. Comp. Biochem. Physiol. A Mol. Integr. Physiol..

[83] Yeltekin A.Ç., Oğuz A.R. (2022). Toxic Effects of Sodium Lauryl Sulfate on Antioxidant Defense System and DNA Damage in Fish Primary Hepatocyte Cultures. Maced. Vet Rev..

[84] Walker C.H., Sibly R.M., Sibly R.M., Peakall D.B. (2005). Principles of Ecotoxicology.

[85] Martins M.F., Costa P.G., Bianchini A. (2022). Maternal Transfer of Pharmaceuticals and Personal Care Products in the Brazilian Guitarfish *Pseudobatos horkelii*. Environ. Adv..

[86] Martins M.F., Costa P.G., Bianchini A. (2021). Maternal Transfer of Polycyclic Aromatic Hydrocarbons in an Endangered Elasmobranch, the Brazilian Guitarfish. Chemosphere.

[87] Martins M.F., Costa P.G., da Guerreiro A.S., Bianchini A. (2023). Consequences of Prenatal Exposure to Contaminants in Elasmobranchs: Biochemical Outcomes during the Embryonic Development of *Pseudobatos horkelii*. Environ. Pollut..

[88] Desbiolles F., Malleret L., Tiliacos C., Wong-Wah-Chung P., Laffont-Schwob I. (2018). Occurrence and Ecotoxicological Assessment of Pharmaceuticals: Is There a Risk for the Mediterranean Aquatic Environment?. Sci. Total Environ..

[89] Feo M.L., Bagnati R., Passoni A., Riva F., Salvagio Manta D., Sprovieri M., Traina A., Zuccato E., Castiglioni S. (2020). Pharmaceuticals and Other Contaminants in Waters and Sediments from Augusta Bay (Southern Italy). Sci. Total Environ..

[90] Yuan M., Faggio C., Perugini M., Aliko V., Wang Y. (2023). Editorial: Pharmaceuticals, Personal Care Products and Endocrine Disrupting Chemicals: The Physiological Consequences of Exposure to Pollutants in Aquatic Animals. Front. Physiol..

[91] Gelsleichter J., Szabo N.J. (2013). Uptake of Human Pharmaceuticals in Bull Sharks (*Carcharhinus leucas*) Inhabiting a Wastewater-Impacted River. Sci. Total Environ..

[92] Corsolini S., Guerranti C., Perra G., Focardi S. (2008). Polybrominated Diphenyl Ethers, Perfluorinated Compounds and Chlorinated Pesticides in Swordfish (*Xiphias gladius*) from the Mediterranean Sea. Environ. Sci. Technol..

[93] Triassi M., Nardone A., Giovinetti M.C., De Rosa E., Canzanella S., Sarnacchiaro P., Montuori P. (2019). Ecological Risk and Estimates of Organophosphate Pesticides Loads into the Central Mediterranean Sea from Volturno River, the River of the “Land of Fires” Area, Southern Italy. Sci. Total Environ..

[94] Comoretto L., Chiron S. (2005). Comparing Pharmaceutical and Pesticide Loads into a Small Mediterranean River. Sci. Total Environ..

[95] Stara A., Kubec J., Zuskova E., Buric M., Faggio C., Kouba A., Velisek J. (2019). Effects of S-Metolachlor and Its Degradation Product Metolachlor OA on Marbled Crayfish (*Procambarus virginalis*). Chemosphere.

[96] Sánchez-Bayo F. (2012). Insecticides Mode of Action in Relation to Their Toxicity to Non-Target Organisms. J. Environ. Anal. Toxicol..

[97] Bado-Nilles A., Gagnaire B., Thomas-Guyon H., Le Floch S., Renault T. (2008). Effects of 16 Pure Hydrocarbons and Two Oils on Haemocyte and Haemolymphatic Parameters in the Pacific Oyster, *Crassostrea gigas* (Thunberg). Toxicol. Vitr..

[98] Matozzo V., Zampieri C., Munari M., Marin M.G. (2019). Glyphosate Affects Haemocyte Parameters in the Clam *Ruditapes philippinarum*. Mar. Environ. Res..

[99] Del Giudice G., Prisco M., Agnese M., Verderame M., Rosati L., Limatola E., Andreuccetti P. (2012). Effects of Nonylphenol on Vitellogenin Synthesis in Adult Males of the Spotted Ray Torpedo Marmorata. J. Fish. Biol..

[100] Marsili L., Coppola D., Giannetti M., Casini S., Fossi M.C., Van Wyk J.H., Sperone E., Tripepi S., Micarelli P., Rizzuto S. (2016). Skin Biopsies as a Sensitive Non-Lethal Technique for the Ecotoxicological Studies of Great White Shark (*Carcharodon carcharias*) Sampled in South Africa. Expert Opin. Environ. Biol..

[101] Storelli M.M., Marcotrigiano G.O. (2005). Bioindicator Organisms: Heavy Metal Pollution Evaluation in the Ionian Sea (Mediterranean Sea—Italy). Environ. Monit. Assess..

[102] Salvo A., Potortì A.G., Cicero N., Bruno M., Lo Turco V., Di Bella G., Dugo G. (2014). Statistical Characterisation of Heavy Metal Contents in *Paracentrotus lividus* from Mediterranean Sea. Nat. Prod. Res..

[103] Kemp M., Wepener V., de Kock K.N., Wolmarans C.T. (2017). Metallothionein Induction as Indicator of Low Level Metal Exposure to Aquatic Macroinvertebrates from a Relatively Unimpacted River System in South Africa. Bull. Environ. Contam. Toxicol..

[104] Riani E., Cordova M.R., Arifin Z. (2018). Heavy Metal Pollution and Its Relation to the Malformation of Green Mussels Cultured in Muara Kamal Waters, Jakarta Bay, Indonesia. Mar. Pollut. Bull..

[105] Kwon Y.-K., Jung Y.-S., Park J.-C., Seo J., Choi M.-S., Hwang G.-S. (2012). Characterizing the Effect of Heavy Metal Contamination on Marine Mussels Using Metabolomics. Mar. Pollut. Bull..

[106] Vlahogianni T.H., Valavanidis A. (2007). Heavy-Metal Effects on Lipid Peroxidation and Antioxidant Defence Enzymes in Mussels *Mytilus galloprovincialis*. Chem. Ecol..

[107] Bonsignore M., Salvagio Manta D., Oliveri E., Sprovieri M., Basilone G., Bonanno A., Falco F., Traina A., Mazzola S. (2013). Mercury in Fishes from Augusta Bay (Southern Italy): Risk Assessment and Health Implication. Food Chem. Toxicol..

[108] Reinero F.R., Milazzo C., Minervino M., Marchio C., Filice M., Bevacqua L., Giglio G., Leonetti F.L., Micarelli P., Tripepi S. (2022). Parasitic Load, Hematological Parameters, and Trace Elements Accumulation in the Lesser Spotted Dogfish *Scyliorhinus canicula* from the Central Tyrrhenian Sea. Biology.

[109] Humbert J.-F., Dorigo U. (2005). Biodiversity and Aquatic Ecosystem Functioning: A Mini-Review. Aquat. Ecosyst. Health Manag..

